# Advice-seeking during implementation: a network study of clinicians participating in a learning collaborative

**DOI:** 10.1186/s13012-018-0797-7

**Published:** 2018-07-28

**Authors:** Alicia C. Bunger, Nathan Doogan, Rochelle F. Hanson, Sarah A. Birken

**Affiliations:** 1 College of Social Work, The Ohio State University, 1947 College Road, Columbus, OH 43210 USA; 2 The Ohio Colleges of Medicine Government Resource Center , The Ohio State University, 1070 Carmack Road, Columbus, OH 43210 USA; 3 National Crime Victims Research and Training Center, Department of Psychiatry and Behavioral Sciences, Medical University of South Carolina, 100 Doughty Street, Charleston, SC 29425 USA; 4 Department of Health Policy and Management, Gillings School of Global Public Health, University of North Carolina at Chapel Hill, 1105C McGavran-Greenberg Hall, Campus Box 7411, Chapel Hill, NC 27599 USA

**Keywords:** Learning collaborative, Advice-seeking, Social network analysis, Mental health services

## Abstract

**Background:**

Successful implementation depends on the transfer of knowledge and expertise among clinicians, which can occur when professionals seek advice from one another. This study examines advice-seeking patterns among mental health clinicians participating in learning collaboratives (a multi-component implementation and quality improvement strategy) to implement trauma-focused cognitive behavioral therapy (TF-CBT). We apply transactive memory system theory, which explains how professionals access and retrieve knowledge, to examine factors associated with the evolution of advice-seeking relationships during implementation. Our aim is to unpack learning collaboratives’ mechanisms by investigating how and why advice-seeking networks change, which may help us understand how implementation strategies can best target networks.

**Methods:**

Using social network analysis and a pretest-post-test design, we examined patterns in general and treatment-specific advice-seeking among 146 participants (including five clinical experts) from 27 agencies participating in a regional scale-up of TF-CBT. Surveys were administered in-person at the first and last of three in-person learning sessions (10 months apart) that comprise a core component of learning collaboratives. Participants nominated up to five individuals from whom they seek general and treatment-specific advice. Exponential random graph models (ERGMs) tested the likelihood of maintaining or forming advice-seeking relationships based on indicators of expertise quality, accessibility, need, and prior advice-seeking relationships.

**Results:**

Participants formed or maintained advice-seeking relationships with those who possess perceived expertise (e.g., learning collaborative faculty experts, supervisors, and those with greater field experience than themselves). Participants also tended to seek advice from those within the same organization and with similar disciplinary training, highlighting the importance of expertise accessibility. Prior relationships and network structural features were associated with advice-seeking, indicating that participants built on existing social ties. Advice-seeking did not vary based on participants’ role or experience.

**Conclusions:**

Given the importance of accessible clinical expertise and ongoing supervision for delivering treatment with fidelity, learning collaboratives may support implementation by promoting clinicians’ awareness of and access to others’ expertise, especially those with substantial expertise to share (e.g., faculty experts and supervisors). Future controlled studies are needed to verify the effectiveness of learning collaboratives for building networks that connect clinicians and experts and for improving implementation.

**Electronic supplementary material:**

The online version of this article (10.1186/s13012-018-0797-7) contains supplementary material, which is available to authorized users.

## Background

Implementing evidence-based treatments (EBTs) in health and human service organizations is a complex and knowledge-intensive process requiring clinicians to develop new skills, knowledge, and expertise. The social interactions among clinicians, their supervisors, and other sources of clinical knowledge can serve as conduits for transferring technical information and expertise needed to learn and use an EBT with proficiency [1]. This transfer occurs when professionals seek and share advice with one another. Advice-seeking in this context is a deliberate action where a clinician asks another for expertise around a work-related task, including *general* advice about serving their clients, and *specific* advice for using an EBT. Advice-seeking relationships directly shape clinicians’ EBT use [2] and can be a more powerful influence over work behaviors than other types of social relationships (e.g., friendship) [3].

As professional values, work expectations, and clinical treatments change, clinicians may need new or different types of expertise and thus alter whom they approach [4]. For example, clinicians may build a new relationship and ask advice from a colleague who has demonstrated proficiency in a new EBT; they might ask their trusted mentor or supervisor for advice more frequently than in the past; or they may stop asking advice from a colleague who does not use the new EBT. Advice-seeking patterns (and changes in them) during implementation and other quality improvement initiatives have been documented in studies conducted in public health and healthcare settings. More specifically, these studies suggest that clinicians’ advice-seeking patterns may evolve over time as they learn to implement a new EBP or engage in other quality improvement initiatives [5–7].

However, clinicians may not know who has expertise in the new EBT or have access to them, constraining advice-seeking, and the flow of needed expertise. Large-group interventions that foster interaction and knowledge sharing among professionals have the potential to facilitate advice and expertise sharing needed for implementation. For instance, the Institute for Healthcare Improvement’s Breakthrough Series Learning Collaborative Model has grown in popularity as a multi-organizational, multi-component implementation, and quality improvement strategy [8, 9]. Learning collaboratives have a potential to promote implementation of EBTs in healthcare organizations and systems by fostering shared learning among clinicians and with key content experts. Little is known about how these large-scale implementation initiatives influence clinicians’ advice-seeking patterns and access to expertise. Emerging evidence suggests that clinicians’ advice-seeking and communication patterns change over the duration of learning collaboratives [10, 11] and other large-group quality improvement initiatives [5], as clinicians form new, and even dissolve, pre-existing relationships. But how do these relationships change and do they facilitate access to needed expertise for implementation? The underlying processes that account for these changes and their implications for clinicians’ access to expert clinical advice and implementation are unclear.

To address this gap, this study examined change in professional advice-seeking patterns and factors that underlie the formation and maintenance of these ties among mental health clinicians participating in learning collaboratives to implement trauma-focused cognitive therapy (TF-CBT), an EBT for treating children with trauma-related mental health and behavioral problems [12, 13]. Understanding how clinicians seek and access expert advice over time has a potential to unpack potential mechanisms that underlie the evolution of advice-seeking networks and inform whether refinements are needed for learning collaboratives and other networked strategies (that may leverage or modify social networks to promote implementation).

### Facilitating knowledge and expertise sharing for implementation––learning collaboratives

Originally developed by the Institute for Healthcare Improvement to guide quality improvement initiatives in healthcare systems, the learning collaborative has been widely used within behavioral health-care to improve care processes (e.g., improving family engagement or reducing wait times), as well as to promote the implementation of evidence-based practices [14, 15]. Learning collaboratives emphasize shared learning by bringing multidisciplinary teams together from several organizations or units over 8–12 months under the guidance of faculty experts. Although the specific components may vary, the typical structure of a learning collaborative involves teams that participate in in-person training, or *learning* sessions, where faculty experts lead didactic sessions, group discussion, and skill building activities; interspersed action periods at home agency where teams implement the new practice using plan-do-study-act cycles; as well as conference calls, coaching, site visits, and regular monitoring of performance data to support skill acquisition and implementation over the duration of the learning collaborative.

Prior studies have indicated that learning collaborative participants especially appreciated the opportunity to learn from faculty experts and other participants [16–18]. Given the emphasis on intensive interactions and shared learning, learning collaboratives and other large-group interventions have strong potential for altering social networks among participants [19–21]. Evidence suggests that in fact, participants’ networks change during, or in response to large-group interventions. For instance, Yousefi-Nooraei and colleagues found that over a 22 month organizational initiative to promote evidence-informed decision making within public health departments, professionals’ networks changed - those engaged in the intervention strengthened ties with one another, producing clusters of engaged professionals [7]. In addition, earlier descriptive findings published from the present study also suggested that the advice-seeking network among mental health clinicians became more centralized around key faculty experts, as clinicians increased their ties to learning collaborative leaders over time [10]. Also internal communication among organizational team members strengthened [11]. Thus, learning collaboratives and other large-group interventions have potential to alter advice-seeking and other social networks - however, exactly how and why participants form new advice-seeking ties, and the implications for implementation remain unclear.

### What drives professional advice-seeking?

To understand what drives changes in advice-seeking relationships among learning collaborative participants, this study draws on transactive memory systems theory [22]. Transactive memory systems theory is based on the notion that a group is more knowledgeable than an individual and explains how knowledge sharing networks form, evolve, and influence group performance [23, 24]. According to transaction memory systems theory, specialized knowledge, expertise, and skills are distributed across individuals within groups; to make the most of complementary capacities within a group, members must have a shared and accurate sense of “who knows what” and the ability to access expertise [25]. Thus, free flowing information within the network is a function of an individual’s ability to recognize and identify others with expertise, the decision to seek and retrieve that expertise, adjusting perceptions of “who knows what”, and deciding whether and with whom that newly acquired information should be shared [26]. Within a knowledge network, ties between individuals facilitate awareness of others’ expertise and the flow of information. Therefore, the advice seeker, the advice source, and their relationships matter. These ties can form naturally over time, but can also be stimulated by large-group interventions, which are deliberate organizational or system development interventions that engage whole teams, organizations, or systems in dialog, learning, and change [19]. A variety of models are commonly used in public and private management and include participatory methods for work redesign and EBT implementation (e.g. Search Conferences, World Cafes, large-group training) [27–29].

We posit that learning collaboratives are one such large-group intervention that can alter advice-seeking networks. Consistent with transactive memory systems theory, our working hypothesis is that learning collaboratives, and other types of group trainings, stimulate formation of knowledge and advice-seeking networks by offering opportunities for intensive interactions [30]. During their interactions, clinicians’ become more aware of others’ expertise (the knowledge recognition process described in transactive memory systems theory). In addition, in person, phone, and web meetings with other clinicians and the local experts facilitate regular access to these individuals, and encourage clinicians to seek and retrieve new advice and expertise. As a result of heightened awareness of other’s expertise, and opportunities to interact, clinicians are able to locate and access assistance when they need advice. Therefore, we expect that general and TF-CBT-specific advice-seeking during a learning collaborative evolves in predictable ways: professionals seek advice and expertise from others when they are aware of and can easily access their colleagues’ expertise [22, 31]. Thus, advice-seeking is likely to be determined by characteristics of the advice source (the perceived quality of their expertise and their accessibility), the advice-seeker (their need for expertise), and their relationship (whether there are existing relationships) [32].

### Characteristics of the advice source

#### Expertise quality

First, professionals are likely to seek advice from colleagues who are known, perceived as competent, and whom they believe can offer high quality, appropriate expertise in a given situation [32]. In fact, perceived quality of the advice sources is one of the most salient motivators for advice-seeking [32, 33]. Accordingly, professionals with senior positions, extensive experience, and specialized training or credentials in a particular EBT are often sought after for advice and assistance [34, 35]. Title and role labels provide clear signals around key expertise sources in work settings [36]. For example, within learning collaboratives, there are faculty experts leading the learning and coaching sessions, and other participants are supervisors or senior leaders denoting some authority, experience, and expertise. Thus, participants may be more likely to maintain existing relationships or form new advice ties with individuals perceived as experts (e.g. faculty expert, senior leader, supervisor).

In addition, shared learning activities during learning collaboratives provide opportunities for participants to get to know their teammates better, as well as their perspectives, thoughts, experiences, and skills, and to identify colleagues with valuable expertise. More seasoned professionals, especially those that have received prior training in the EBT, often have extensive practical experience applying the model in practice, observing clients’ responses, and working through implementation and clinical barriers. This experience and expertise may be especially helpful to others who have questions or concerns about how to use a new treatment with a particular client population. Consequently, participants might maintain relationships or form new ones with individuals who are perceived as experts. Thus, we hypothesize that the likelihood of maintaining or forming advice-seeking relationships will be positively associated with the perceived quality of an advice source’s expertise (as indicated by their role, field experience, and prior training in TF-CBT) (H1).

#### Accessibility and proximity

For expertise quality to drive advice-seeking within and across organizations, clinicians must have enough information about their colleagues’ knowledge and skills to inform their advice-seeking decisions. However, especially in busy community mental health settings, clinicians rarely have time to systematically inventory “who knows what” about a treatment and its intervention, or wait for advice that might not be offered, useful, or without social costs. In fact, clinicians are unlikely to ask for assistance from those who are perceived as distant, unapproachable, or unable to respond [32]. Consequently, and similar to other choices made with incomplete information, clinicians may satisfice by choosing an advice source who is known, in close proximity, and accessible [31, 37, 38]. In fact, accessibility is one of the most consistent and important determinants of advice-seeking [31, 39, 40]. Learning collaboratives bring clinicians into closer proximity to expertise during learning sessions, structured coaching calls, group work among implementation teams, conference calls with other teams, and site-visits with faculty experts. Thus, learning collaboratives might facilitate access to local expertise and, therefore, promote advice-seeking among participants who work at the same organization or participate in the same learning collaborative cohort. In a similar vein, colleagues are likely to rely on advice from others in close social proximity (e.g. being trained in the same discipline), or who are socially accessible [31]. Participants may find it easier to ask for help or information from a colleague with comparable training because they share common professional values, norms, language, and practice skills [37]. In other words, belonging to similar groups, and sharing a common group identification can reduce cognitive barriers associated with asking for help [33]. As a result, over the duration of a learning collaborative, participants might seek advice on treatment from a colleague in the same discipline. Thus, we hypothesize that the likelihood of forming or maintaining advice-seeking relationships is positively associated with shared physical or social proximity (similar disciplinary training) of the advice-seeker and source (H2).

### Needs of the advice seeker

In addition to characteristics of the advice source, advice-seeking depends on the needs of the individual who is looking for help. Professionals seek advice when it is needed and valued; when individuals encounter a problem that they do not have the knowledge or comfort to solve [38]. Thus, professionals who are newer to the field, or have a little training or experience in a particular EBT, may seek advice more often than those who are more experienced [31, 32]. Also, professionals in front-line positions in the organizations may have greater advice needs than supervisors or senior leaders because they are directly responsible for delivering a new EBT. Even experienced clinicians may consult others often for technical information or feedback until they feel comfortable using the new treatment. As a result, front-line clinicians may have more robust advice networks than their supervisors or agency leaders who may not be directly using the new treatment. Thus, changes in advice-seeking over the duration of a learning collaborative may vary based on an individuals’ role in the organization. Thus, we hypothesize that front-line workers (clinicians) will be more likely to form or maintain advice-seeking relationships than supervisors or senior leaders (H3).

### The role of existing relationships and network structure

Finally, advice-seeking is often conditioned by existing social networks and structural properties of these ties (key terms are defined in Table 1) [41–43]. When confronted with a new problem, professionals are likely to turn to colleagues they know and trust [35]. As a result, new professional advice-seeking patterns often build on prior relationships (direct relationships with others), where professionals turn to existing mentors who have shared other types of advice previously. In the context of a learning collaborative, where participants are learning to implement EBTs, participants might solicit advice on the new specific treatment from someone who has previously advised them on general treatment or practice. Relationships based on multiple types of advice are considered *multiplex* and may be associated with formation and maintenance of advice-seeking ties. Similarly, participants might strengthen their relationships with colleagues who have asked them for advice. Professionals who share their expertise often expect their partner to share in return [32, 42, 44]. Therefore, learning collaborative participants might develop new advice-seeking patterns in *reciprocity* with existing advice sources. These reciprocated ties are stronger than one-way relationships, and during a learning collaborative, advice-seeking may become increasingly reciprocal as participants rely on one another.

**Table 1:** Key terms and definitions

Term	Definition
Network	A system structure that arises from a pattern of interactions/ties/relationships among a set of actors.
Advice-seeking	A deliberate action where a clinician asks another for expertise around a work-related task, such as using an EST. In this study, advice-seeking is the type of tie examined among participants.
Multiplex	Ties between two individuals that are based on more than one type of relationships (e.g., seeking advice on a new EST and seeking advice on treating children exposed to trauma).
Reciprocity	The tendency to form mutual advice-seeking relationships where participant A seeks advice from participant B, and participant B seeks advice from participant A.
Transitivity	The tendency for two participants who seek advice from one another to share a mutual third party partner (triadic pathways). “The friend of a friend is my friend.” If participant A seeks advice from participant B, and participant B seeks advice from participant C, then A is also likely to seek advice from participant C.

In professional contexts, individuals might connect their colleagues to a trusted mentor for additional advice. Having common third-party colleagues can facilitate advice sharing and knowledge transfer [33, 42]. For example, a clinician who provides advice to a colleague might also connect her colleague to one of her mentors. Thus, two participants who share advice, also share a mutual third party mentor. This tendency toward triadic pathways of advice-seeking (“a friend of a friend is a friend”) is called *transitivity* and creates tighter clusters among participants. As participants in the learning collaborative learn and locate expertise, they may become increasingly connected through these third-party ties. Based on this evidence, we hypothesize that participants will be more likely to form and maintain advice-seeking ties with colleagues with whom they have existing relationships either directly (by building multiplex relationships or in reciprocity) or through a mutual third party (transitivity) (H4).

## Methods

### Study context and design

This study was conducted in the context of a regional initiative to implement TF-CBT, within a large county in the Midwest (USA). Funded through a county-based sales tax levy, a university-based mental health agency was contracted to design and lead the learning collaborative. Five therapists from the university-based mental health agency served as faculty experts––TF-CBT treatment developers previously trained all five therapists, and three completed the “Train the Trainer” program. The learning collaborative model was based on the National Center for Child Traumatic Stress (NCCTS) Learning Collaborative which integrates the core IHI Breakthrough Series Collaborative components with clinical training and coaching [9, 17]. Participation in the learning collaborative was open to the 44 agencies funded through the county sales tax levy. Participating agencies selected implementation teams comprised of three to ten staff members including a senior administrative leader, a clinical supervisor, and front-line clinicians. Of those invited, 32 agencies volunteered to participate and selected 206 staff to attend. To accommodate the demand (given the availability of funding, time, space, and faculty), four learning collaboratives were conducted (with about 30 participants in each), beginning in April 2011 and ending in September 2012.

The learning collaborative model used in this study has been previously specified [10]. Briefly, this model offered several opportunities for clinicians to interact with other clinicians and faculty experts. Active learning sessions and cross-site conference calls (where there was discussion of shared experiences/strategies, small group breakouts to allow more face-to-face time within each cohort, and behavioral rehearsal activities) were intended to promote participants’ awareness of and access to their external colleagues’ expertise. Collective trouble-shooting and refinement during Plan-Do-Study-Act cycles (small iterative tests of change) were intended to promote clinicians’ access and use of their internal colleagues’ expertise. Finally, coaching calls and on-site visits were intended to build awareness of and access to faculty experts’ knowledge and skills.

To examine changes in social ties, the external research team designed a pre/post-test assessment and administered it during the in-person learning sessions.

### Participants and data collection

Across the four LC cohorts, 145 individuals from 32 agencies completed the LCs (70% completion). Of these individuals, 126 (86.9%) voluntarily responded to two surveys administered in person by research staff during the first learning session (time 1) and again approximately 10 months later during the last learning session (time 2) of each learning collaborative.

### Measures

#### Advice-seeking (dependent variables)

Two types of advice-seeking relationships were measured using an ego-network approach [45]. At both time points, participants were asked to nominate up to five individuals to whom they turned to in the past 6 months for (1) general advice about youth with a trauma history and (2) specific advice about TF-CBT. We refer to the nominator as ego and the nominees as alters. For each alter nominated, participants reported the name and organizational affiliation. This process identified 422 individuals. Because this study focused on development of advice-seeking relationships among learning collaborative participants, our analysis is restricted to the ties among the 126 participants and five faculty experts (131 total).

#### Professional characteristics

We measured professional characteristics including organization, disciplinary training (e.g., social work, counseling, psychology), and role on the implementation team (senior leader, supervisor, or clinician). General experience in the field and experience working at the current agency were measured with two categorical survey items, where responses included from 0 to 6 months, 6–11 months, 1–3 years, 3–5 years, and more than 5 years. In addition, because some participants may have come to the learning collaborative with some knowledge of TF-CBT, respondents were asked to indicate whether they received prior training on TF-CBT (binary, where 1 = yes).

### Analysis

In preparation for analysis, networks were visualized and several general descriptive network metrics were calculated for each advice-seeking network at both time points (see Additional file 1). Exponential random graph models (ERGMs) were used to test hypotheses about formation and maintenance of the general and specific advice-seeking ties at the end of the learning collaborative by conditioning on the ties present at the first observation [46]. This lagged ERGM approach has been used in other network studies with only two measured time points [47]. ERGMs are models of the probability of, in our case, advice-seeking ties among a set of nodes. From a practical standpoint, model fitting and interpretation with ERGMs proceeds similarly to an analysis using logistic regression. Both are multivariable approaches. Two major differences are that ERGMs (1) allow the researcher to control for the dependencies in relational data that may otherwise cause issues for estimation or inference and (2) offer numerous possibilities to test hypotheses about the mechanisms likely to underlie the formation or evolution of a network that has been measured at a particular moment in time. The ERGMs we fit included terms that allowed the model to adjust the odds of the presence of ties depending on (1) the presence of other ties in the network (e.g., those that are reciprocal or transitive), (2) characteristics of the advice seeker, source, or (3) some function of the characteristics of both (e.g., employment at the same agency). Since the primary interest was in describing how the network changed from the beginning to the end of the learning collaborative, we modeled the time 2 network as a function of terms like those described above *and* a term that adjusted the odds that a tie existed at time 1 (i.e., a dyadic covariate). We refer to these model terms as lag terms. Terms included the following:

#### Indicators of expertise quality (H1)

Five terms reflected advice quality, including whether the potential advice source was a senior leader, supervisor, faculty expert, had prior TF-CBT training, and whether the potential advice source had more experience in the field than the advice-seeker (participant).

#### Indicators of advice accessibility (H2)

Three terms reflect advice accessibility relative to the advice-seeker (H2), including whether the advice-seeker and their potential advice source were from the same organization, in the same learning collaborative cohort, and trained in the same discipline.

#### Characteristics of the advice-seeker (participant) (H3)

Three terms reflected the advice-seeker’s potential need for advice, including their role (where clinician served as the referent), prior training in TF-CBT, and experience in the field.

#### Existing relationships (H4)

These terms included the presence of general and specific advice-seeking ties at time 1 (two lag terms) and reciprocity (the dependence between the presence of a tie from an individual A to another B and the presence of a tie from B to A). We also included two terms to account for transitivity (if participant A seeks advice from participant B, and participant B seeks advice from participant C, participants A and C are also likely to have a tie). *Geometrically weighted edgewise shared partners* (GWESP) term accounts for the number of partners that a participant and advice source share. Theoretically, each additional shared tie does not increase the probability of a tie between A and B equally, this term contributes positively to the probability of a tie between two actors based on increasing shared partners, but does so in a decreasing way. We also included a 3-cycle term, which accounts for shared partners but only for cyclic relationships where A seeks advice from B, B seeks advice from C, and C seeks advice from A. These cycles are often indicators of egalitarian (non-hierarchical) knowledge sharing patterns.

Negative parameter estimates represented odd adjustments in the direction of tie dissolution if the tie was present at time 1, or non-creation (if it was not), and positive estimates represented adjustments in the direction of either tie creation or tie maintenance. Two fit indices, Akaike and Bayesian Information Criterion (AIC and BIC), were calculated for each model. The models were built in a step-wise fashion of increasing complexity. (Our results and relative fit statistics are detailed in Additional file 2). We report the most complete model (model 4) that includes all of our terms.

## Results

### Participant characteristics

Participant characteristics are displayed in Table 2. Two-thirds (67%) of participants provided direct clinical services, 22% were supervisors, and 10% were senior leaders. All participants held a master’s degree or higher in social work (54%), counseling (17%), psychology (12%), or other discipline (16%). A majority practiced in children and family service settings for more than 5 years (65%), although 44% worked in their current position for less than 1 year.

**Table 2:** Participant characteristics (*n* = 126)

	Number	Percent
Role		
Senior leader	13	10.32
Supervisor	28	22.22
Clinician	85	67.46
Education		
Bachelor’s degree	2	1.59
Master’s degree	116	92.06
Doctoral degree or equivalent	8	6.35
Discipline		
Social work	68	53.97
Counseling	22	17.46
Psychology	15	11.9
Other (education, medicine, addictions)	21	16.67
Experience in the field		
0–6 months	1	0.79
6–11 months	6	4.76
1–3 years	18	14.29
3–5 years	19	15.08
more than 5 years	82	65.08
Experience in current job		
0–6 months	27	21.43
6–11 months	28	22.22
1–3 years	22	17.46
3–5 years	17	13.49
more than 5 years	32	25.4
Learning collaborative cohort		
1	41	32.54
2	22	17.46
3	39	30.95
4	24	19.05
Prior TF-CBT training		
Yes	75	60

### Factors associated with the formation/maintenance of advice-seeking ties

Final results of the full ERGM models are displayed in Table 3. Interpretation of the coefficients were, as always in multivariable models, taken to be conditional on all other model terms, including the lagged terms (representing time 1 ties). Thus, where the model implies high-tie probabilities, advice-seeking ties were likely to have been maintained from time 1 or newly created at time 2. Similarly, where the model implies low-tie probabilities, advice-seeking ties that were not present at time 1 were likely to be maintained in their null state at time 2 or removed if they were present at time 1.

**Table 3:** ERGM results––factors associated with formation and maintenance of advice-seeking ties

Characteristic	Feature of	General advice-seeking		Specific advice-seeking	
		est	se	est	se
*Expertise quality* (*H1*)					
Senior lead	Advice source	0.321	(0.301)	0.187	(0.370)
Supervisor	Advice source	0.822*	(0.226)	1.092*	(0.223)
Faculty expert	Advice source	4.185*	(0.305)	4.242*	(0.305)
Prior training	Advice source	0.146	(0.210)	0.170	(0.193)
Alter higher experience	Dyad	0.245	(0.234)	0.644*	(0.208)
*Accessibility* (*H2*)					
Same agency	Dyad	2.818*	(0.249)	2.683*	(0.273)
Same cohort	Dyad	0.265	(0.194)	0.256	(0.215)
Same field	Dyad	0.329*	(0.166)	0.372*	(0.187)
*Expertise need* (*H3*)					
Senior leader	Advice seeker	− 0.381	(0.306)	− 0.288	(0.306)
Supervisor	Advice seeker	− 0.072	(0.233)	0.075	(0.226)
Prior training	Advice seeker	− 0.293	(0.174)	− 0.200	(0.181)
Experience	Advice seeker	0.116	(0.098)	0.053	(0.106)
*Existing relationships* (*H4*)					
General ties lag	Dyad	1.996*	(0.244)	1.515*	(0.259)
Specific ties lag	Dyad	0.817*	(0.213)	1.379*	(0.340)
Reciprocity	Dyad	2.083*	(0.353)	1.213*	(0.420)
Transitivity	Triad	0.624	(0.636)	0.991*	(0.119)
gwesp.alpha	Triad	2.164*	(0.149)	0.847*	(0.115)
3-cycles	Triad	− 0.988*	(0.274)	− 0.876*	(0.266)
Edges (density)		− 7.078*	(0.497)	− 6.887*	(0.520)

*p < .05

#### H1: expertise quality

Hypothesis 1 was partially supported. During the learning collaborative, advice-seeking ties were more likely to be formed or maintained with a faculty expert or supervisor. Participants tended to form or maintain general and specific advice-seeking relationships with learning collaborative faculty experts (*b* = 4.19, *SE* = .31, *p* < .05; *b* = 4.24, *SE* = 3.05, *p* < .05, respectively) and with others in supervisory roles (*b* = .822, *SE* = .23, *p* < .05; *b* = 1.09, *SE* = .22, *p* < .05, respectively). Participants were likely to form and maintain specific advice about TF-CBT from individuals with more field experience (*b* = .64, *SE* = .21, *p* < .05), but not general advice. Having prior TF-CBT training or the role of the senior leader was not associated with forming or maintaining either type of advice-seeking tie.

#### H2: accessibility and proximity

Hypothesis 2 was supported. Being located within the same organization was associated with the formation or maintenance of general (*b* = 2.82, *SE* = .25, *p* < .05) and TF-CBT-specific advice-seeking (*b* = 2.68, *SE* = .27, *p* < .05). Similar disciplinary training was associated with formation or maintenance of general (*b* = .33, *SE* = .17, *p* < .05) and specific advice-seeking (*b* = .37, *SE* = .19, *p* < .05), although being in the same learning collaborative cohort was not significant.

#### H3: needs of advice-seeker

Hypothesis 3 was not supported. None of the indicators of the advice seekers’ need for expertise were associated with formation/maintenance of either advice-seeking tie.

#### H4: role of existing relationships and network structure

Hypothesis 4 was supported. The presence of a general advice-seeking tie at time 2 was significantly related to the presence of the same general advice-seeking tie at time 1 (*b* = 2.0, SE = .24, *p* < .05) and to the corresponding TF-CBT-specific advice-seeking tie at time 1 (*b* = 1.38, SE = .34, *p* < .05). Likewise, the presence of a specific advice-seeking tie at time 2 was significantly related to the presence of the same specific advice-seeking tie at time 1 (*b* = 1.52, SE = .26, *p* < .05) and the general advice-seeking tie at time 1 (*b* = .82, SE = .21, *p* < .05). Reciprocity was positively and significantly associated with the formation/maintenance of general (*b* = 2.1, SE = .35, *p* < .05) and specific advice-seeking ties (*b* = 1.2, SE = .42, *p* < .05) indicating that both forms of advice-seeking tend to be reciprocated. Transitivity was positively associated with the formation/maintenance of TF-CBT-specific advice-seeking (*b* = 1.0, SE = .12, *p* < .05); these results indicate that individuals seek advice from those who provide advice to their other advice sources (e.g., the mentor of my mentor becomes my mentor as well). Transitivity was not associated with general advice-seeking. Also, the effect of three cycles, an additional triadic dependence term, was negative and significant in both the general (*b* = −.99, SE = .28, *p* < .05) and specific advice-seeking models (*b* = − .88, SE = .27, *p* < .05), suggesting a hierarchical pattern of advice-seeking.

## Discussion

As clinicians adopt and implement EBTs, their knowledge and skill needs might change, necessitating new relationships with colleagues who have relevant general and treatment-specific expertise. Drawing on transactive memory systems theory, strategies should help clinicians develop a shared and accurate sense of who is an expert in a particular EBT and promote access to them [25]. Our results suggest that learning collaboratives may do just this. Mental health clinicians participating in a learning collaborative to implement TF-CBT formed or maintained advice-seeking relationships with those with treatment expertise (especially faculty experts and supervisors) and who were easily accessible (e.g., located in the same agency). However, clinicians’ pre-existing social relationships determined with whom they built or maintained advice-seeking ties, which, in some situations, could constrain clinicians’ access to high-quality expertise. These findings have implications for learning collaborative model refinements.

### High-quality expertise

Our findings that expertise quality and accessibility drove the formation of advice-seeking relationships are consistent with other studies rooted in transactive memory systems theory [22, 31, 32]. Consistent with our first hypothesis, clinicians formed and maintained general and TF-CBT-specific advice-seeking relationships with those who were faculty experts, supervisors, or who had more field experience than they did. These clinical leaders had expertise and experience to diffuse through clinicians’ advice networks. Faculty experts were trained TF-CBT clinicians; they introduced the treatment, led the learning sessions, and provided hands-on coaching and consultation––strategies associated with implementation effectiveness [48]. Given how clinicians formed and sought advice from expert faculty over the duration of the learning collaboratives, faculty members likely had more clinicians seeking their advice over time. In this study, five faculty experts were available to the 126 completers. While it is unclear whether five faculty members offered sufficient capacity for meeting participants’ advice needs, the number of faculty experts available to clinicians over the duration of the learning collaborative may be an important consideration for future learning collaborative or implementation initiatives.

Within organizational teams, senior leaders were less sought-after advice sources perhaps because these individuals are responsible for administration and management and may not serve in clinical capacities. However, clinicians formed and maintained advice-seeking relationships with their supervisors. Supervisors are critical to implementation. As middle managers, they diffuse information, synthesize information, mediate between strategy and day-to-day activities, and sell implementation [49, 50]. By sharing general and specific advice, supervisors may have engaged in each of these activities. They might diffuse information by answering questions about TF-CBT, synthesize information by helping clinicians apply TF-CBT with their clients, mediate between organizational goals and clinicians’ TF-CBT use by translating the key TF-CBT components into concrete tasks, and promote implementation by encouraging clinicians’ TF-CBT use. With this in mind, learning collaboratives might emphasize supports for supervisors to help them engage in these activities in a way that is likely to promote desired outcomes.

### The importance of accessibility and existing relationships

Consistent with our second hypothesis, clinicians tended to seek advice from their colleagues in the same agency or with similar disciplinary training. However, being in the same learning collaborative cohort did not seem to influence clinicians’ advice-seeking patterns, although the cohort structure was intended to improve access to others’ knowledge and expertise. Other studies have demonstrated that social and physical accessibility are important considerations in diffusing knowledge and expertise [32], as clinicians are likely to turn to colleagues who they are close to physically (e.g., down the hallway) [22, 51] and believe will provide reliable and understandable information [52]. Clinicians might have prioritized advice from colleagues from the same agency or disciplinary background over other participants in the same learning collaborative cohort. Related to these findings, and consistent with our fourth hypothesis, clinicians formed and maintained advice-seeking ties with colleagues with whom they were already directly or indirectly connected. Clinicians asked for TF-CBT advice from colleagues who previously offered general clinical advice and vice versa. Likewise, clinicians reciprocated advice-seeking and connected their colleagues to their own advice sources (transitivity). These findings indicate that clinicians build on existing networks when forming new relationships and are consistent with other studies demonstrating that network evolution is driven by strong endogenous effects [53, 54].

Together, these findings illustrate how clinicians’ developed and maintained advice-seeking ties with colleagues who are “close” and emphasize the importance of accessibility when seeking advice in the workplace. However, the most accessible colleagues might not always have the most relevant, appropriate, or advanced expertise; likewise, the most appropriate experts might not be the most accessible. Relying on colleagues who are most accessible, instead of those who are most knowledgeable could constrain the information, knowledge, and support that clinicians’ receive [38], limiting their proficient use of an EBT. With this in mind, learning collaboratives and other large-group interventions could emphasize strategic networking, between supervisors and the faculty experts [48]. Our results demonstrate that (1) clinicians sought advice from their supervisors, and (2) there were transitivity effects, which could suggest that it is possible that clinicians seek advice from their supervisors’ advice source. Thus, building and strengthening relationships between supervisors and faculty experts a has potential to enhance front-line clinicians’ access to faculty experts. In other words, if a supervisor is unable to provide or is unsure of advice needed, they can turn to a faculty expert, or connect their worker to her or him. At the same time, these models might scale back broad networking components among clinicians across agencies which could be counterproductive if they end up strengthening ties with individuals who do not have strong skills or knowledge in the EST.

### Participants seek advice in similar ways

Contrary to our expectations, clinicians, supervisors, and senior leaders sought advice in comparable ways. This suggests that participants, regardless of their role in their organization, need and rely on advice when implementing a new EBT. Also, despite having some prior knowledge of the EBT (and potentially valuable advice to share), those with prior TF-CBT training were not more likely to be tapped as advice sources, or need less advice than those without prior training. It may be that clinicians preferred advice from faculty experts and supervisors over their peers with prior exposure to TF-CBT. Together, these findings suggest that future learning collaboratives should involve multiple organizational stakeholders, regardless of their prior training, and help connect them to available expertise.

### Limitations and future directions

The findings should be interpreted in light of limitations that have implications for research on networks, learning collaboratives, and implementation. First, this study was conducted in one geographic region with a strong collaborative history [54]. Given the importance of existing relationships to network evolution, generalizability might be limited to similar service delivery systems. Second, we measured social networks based on self-report of two advice-seeking ties (general and TF-CBT specific) and did not capture implementation-related advice. Our measures also did not capture friendship ties, which influence clinicians’ attitudes toward an EBT, but are more stable than advice ties (and less likely to change in response to learning collaboratives) [4]. Third, the size of our learning collaborative cohorts (about 30 participants) is both larger and smaller [55, 56] than cohorts reported in other learning collaborative studies. Large groups may experience more growth in social networks than small groups [11, 57]. However, we do not anticipate that the mechanisms guiding advice-seeking (which we focus on in this study) vary based on cohort size, although this should be confirmed in a future study. Fourth, without a comparison or control group, we cannot determine whether the change in advice-seeking was driven by the learning collaborative, the need to learn TF-CBT, or other random factors. Future controlled studies are needed to verify the effectiveness of learning collaboratives on advice network evolution, implementation outcomes, and clinical outcomes. A three-wave design could allow for a quasi-experiment where the first period (T1–T2; pre-collaborative) could serve as a baseline picture of network evolution, and the second (T2–T3; during and post collaborative) could be used to examine how the network evolution process changed with the learning collaborative. Such an experiment would be ideally modeled using stochastic actor-based models for network dynamics [58].

## Conclusions

Learning collaboratives and other quality improvement collaborative models have a potential to support implementation by altering the social networks that diffuse expertise, knowledge, and skill for using an EBT, but the change mechanisms were unknown [59, 60]. In this study, participants formed and maintained advice-seeking relationships with faculty experts and supervisors, who were experienced and well positioned to support implementation. Viewed through the lens of transactive memory systems, our results suggest that learning collaboratives might activate clinicians’ awareness and ability to recognize expertise and retrieve it from accessible sources. This model and other large-group implementation interventions could be streamlined to emphasize network building among clinicians and those with the most relevant, appropriate, and high-quality expertise.

## Additional files

Additional file 1: Description of advice-seeking networks over time (PDF 1793 kb)

Additional file 2: ERGM model-building details (DOCX 37 kb)

## Abbreviations

EBT: Evidence-based treatment
ERGM: Exponential random graph model
GWESP: Geometrically weighted edgewise shared partners
NCCTS: The National Center for Child Traumatic Stress
TF-CBT: Trauma-Focused Cognitive Behavioral Therapy
