# Supplementary material for: Advice-seeking during implementation: a network study of clinicians participating in a learning collaborative
Source: Implement Sci. 2018 Jul 28;13:101. doi: 10.1186/s13012-018-0797-7 (PMC6064109; doi:10.1186/s13012-018-0797-7)
Supplement: Supplementary file 1 — Description of advice-seeking networks over time (PDF 1793 kb) [file 13012_2018_797_MOESM1_ESM.pdf]

Additional file 1

Description of Advice-Seeking Networks Over Time

The *sna* package in R [1]was used to plot the general and specific advice-seeking networks and calculate several global network metrics at both time points: *Isolates* represented the number of individuals who neither sought nor provided advice to other network members and were thus isolated in the network. *Network density* was calculated as the proportion of all possible directed dyadic relationships that were reported by the participants to exist. *Reciprocity*, the proportion of ties that were reciprocated, measured the extent to which advice-seeking tended to be unidirectional (lower values) or mutual (higher values). *Transitivity* was measured as the tendency of participants and their advice givers to agree on the third person from whom to seek advice—an indication that advice-seeking activity depends on existing advice ties. *Centralization* captured the degree to which advice-seeking represented by the network seems to emanate from or converge on a select few individuals versus a more distributed advice-seeking network. The *Jaccard similarity index* represented the amount of change in advice seeking ties over the duration of the learning collaborative. The coefficient ranges from zero (no similarity in ties over time) to 1 (no difference in the ties over time). Finally, the E-I index assessed the proportion of in-group (intra-organizational) to out-of-group (inter-organizational) advice-seeking ties [2]. The index ranges from -1 (all ties are internal to an organization) to +1 (all ties are external), where 0 represents an equal proportion of internal and external ties. To understand changes in internal and external ties among clinicians who participated in the learning collaborative, E-I indices were calculated both with and without the faculty experts.

Metrics for the general and TF-CBT specific advice-seeking networks are displayed in Table 1 and 2, and networks are visualized in Figures 1 and 2. Both networks grew more connected; the number of isolated participants who neither sought nor shared advice declined, and there was a small increase in network density (from 1.4% to 1.8% in the general, and 1.4% to 2.0% in specific advice networks). Relationships strengthened and grew slightly more reciprocal in the general advice-sharing network (from 18.8% to 20.2%), but reciprocity declined in the specific advice network (from 17.2% to 16.6%). Both networks experienced small declines in transitivity (the tendency to seek advice from a colleague's advice source), and increases in the degree to which advice seeking is centralized around one or few actors. The specific advice-seeking network experienced greater increases in centralization (from 3.6% to 11.5%) compared to the general advice sharing network (from 2.1% to 5.9%) suggesting that a few central participants became key TF-CBT advice sources. The Jaccard index, was comparable in the two networks, although higher in the general advice than the specific advice network, suggesting slightly greater stability among general advice ties. The E-I indices for both the general and TF-CBT specific advice seeking networks at baseline (both with and without faculty experts) suggested that the majority of ties were internal, and that clinicians predominantly sought advice from internal colleagues from the same home organization. For both networks, E-I indices increased slightly over time suggesting that clinicians increasingly sought advice from colleagues outside of their organization, although still relied on their home colleagues for advice. This increase was larger when faculty experts were included in calculations (because faculty experts represent external ties).

Note that a descriptive analysis of the composition of participants' ego-networks – the proportion of advice sources who are faculty experts, peers from the home agency, peers at other agencies, and private practitioners – is available elsewhere [3]. Those findings also illustrate how clinicians' significantly increased their ties to faculty experts, decreased ties to private practitioners, but ties to peers at home and in other organizations remained stable.

Table 1. Network Metrics Over Time

	General Advice		TF-CBT Specific Advice	
	G1	G2	S1	S2
Network Size	131	131	131	131
Isolates	10	6	17	3
Density	0.014	0.018	0.014	0.020
Reciprocity	0.188	0.202	0.172	0.166
Transitivity	0.436	0.392	0.460	0.412
Degree Centralization	0.021	0.059	0.036	0.115
E-I Index (without experts)	-.86	-.76	-.84	-.67
Jaccard Index	0.392		0.356	

Table 2. Change in Internal and External Advice Seeking Ties

	General Advice		TF-CBT Specific Advice	
	G1	G2	S1	S2
Without Faculty Experts				
External Ties	14	27	16	40
Internal Ties	192	195	185	205
E-I Index	-.86	-.76	-.84	-.67
With Faculty Experts				
External Ties	19	86	28	116
Internal Ties	215	223	211	236
E-I Index	-.84	-.44	-.77	-.34

Figure 1a. General Advice-Seeking Networks – Time 1 (Letters and numbers represent organizations; boxes [k] indicate supervisors, pluses (+) indicate senior leaders, and boxed stars [*] denote faculty experts)

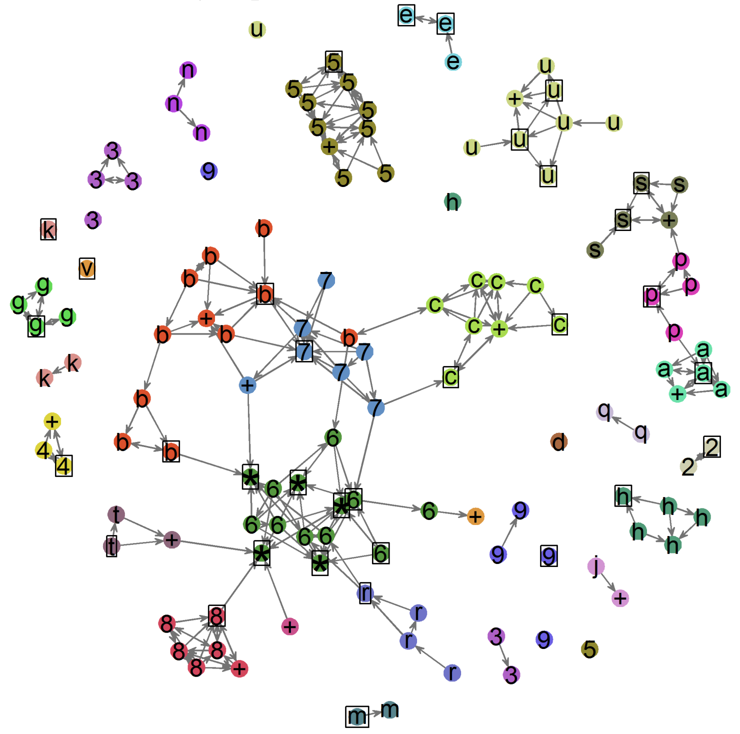

Figure 1b. General Advice-Seeking Networks – Time 2

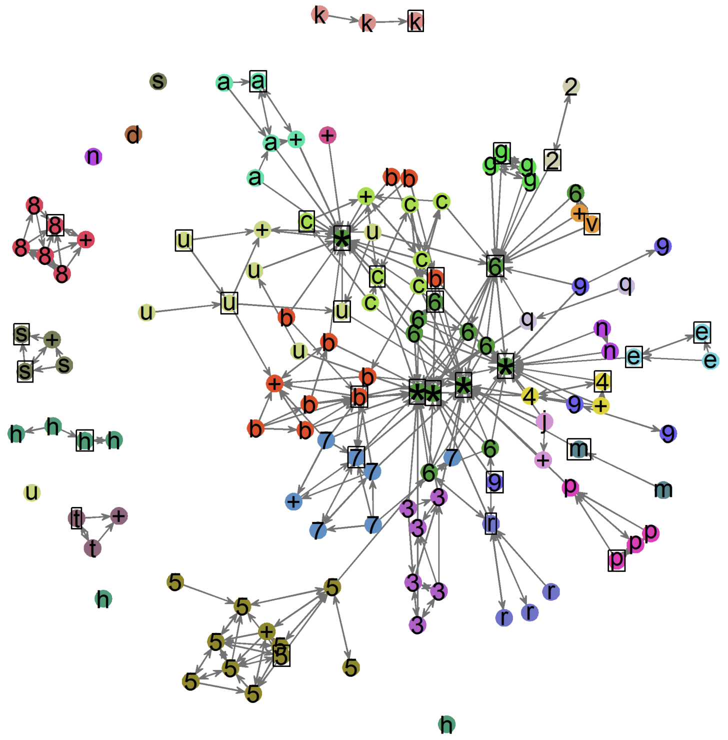

Figure 2a. Specific Advice-Seeking Networks – Time 1 (Letters and numbers represent organizations; boxes [k] indicate supervisors, pluses (+) indicate senior leaders, and boxed stars [*] denote faculty experts)

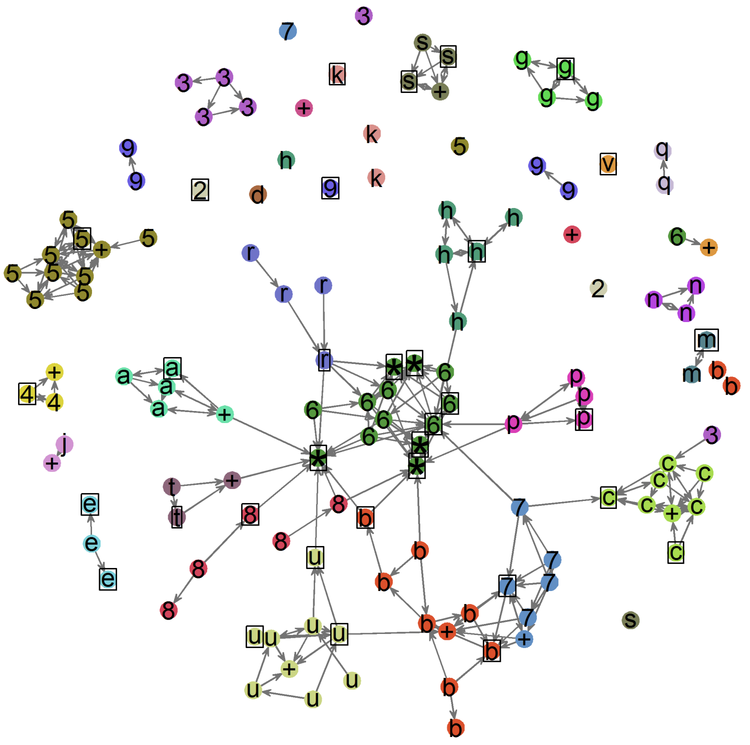

Figure 2b. Specific Advice-Seeking Networks – Time 2

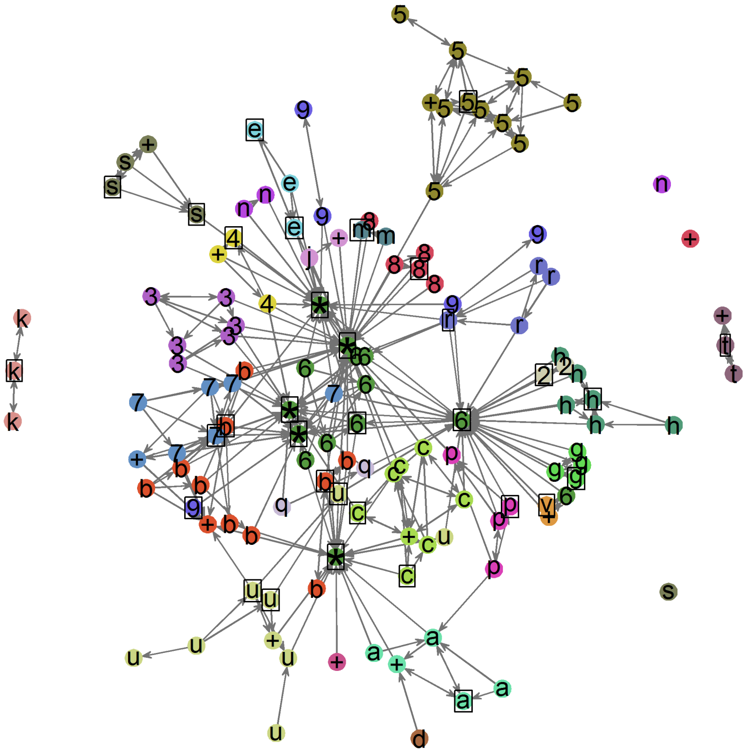

References

1. Butts C. sna: Tools for Social Network Analysis. R package version 2.4. 2016.
2. Krackhardt D, Stern RN. Informal Networks and Organizational Crises: An Experimental Simulation. Soc. Psychol. Q. American Sociological Association; 1988;51:123–40.
3. Bunger AC, Hanson RF, Doogan NJ, Powell BJ, Cao Y, Dunn J. Can Learning Collaboratives Support Implementation by Rewiring Professional Networks? Adm. Policy Ment. Heal. Ment. Heal. Serv. Res. Springer US; 2016;43:79–92.
