# Supplementary material for: Advice-seeking during implementation: a network study of clinicians participating in a learning collaborative
Source: Implement Sci. 2018 Jul 28;13:101. doi: 10.1186/s13012-018-0797-7 (PMC6064109; doi:10.1186/s13012-018-0797-7)
Supplement: Supplementary file 2 — ERGM model-building details (DOCX 37 kb) [file 13012_2018_797_MOESM2_ESM.docx]

**Additional file 2**

ERGM Model-Building Details

To prevent degeneracy, the ERGM models were built in a step-wise fashion:

*Model 1*: First, we modeled general and specific advice-seeking ties based on existing relationships (H 4). These terms included:

- the presence of general advice-seeking ties at Time 1 (lag term) (dyadic term)
- the presence of specific advice-seeking ties at Time 1 (lag term) (dyadic term)
- reciprocity (dyadic term)
- GWESP (triadic dependencies)
- 3-cycles term to control for the tendency of triplets to exhibit cyclic advice sharing (triadic dependencies)

*Model 2*: Next, we added three variables related to advice accessibility relative to the participant (ego) (H2). These terms included whether the clinician and their advice source were:

- from the same organization (dyadic term)
- in the same learning collaborative cohort (dyadic term)
- trained in the same discipline (dyadic term)

*Model 3*: Third, we added five variables related to expertise quality (H1) of the advice source (alter).

- Whether the advice source was a senior leader (alter term)
- Whether the advice source was a supervisor (alter term)
- Whether the advice source was a faculty expert (alter term)
- Whether the advice source was a had prior TF-CBT training (alter term)
- Whether the alter had more years of experience than the ego (dyadic term)

*Model 4*: Our last model added three variables that account the advice seeker’s (ego) need for expert advice (H3):

- role (where clinician served as the referent) (ego term)
- prior training in TF-CBT (ego term)
- experience (ego term)

Full results of each modeling step are contained in Tables 1 and 2 below.

*Model Selection*: AIC/BIC estimate relative fit, adjusting for model complexity, across a series of models, whereby the lowest values indicate optimal fit and parsimony. Thus, these are commonly used for model selection. Model 3 in both the general and TF-CBT specific advice-seeking networks has the lowest AIC and BIC values, suggesting the best fit. None of our advice-seeker variables were significantly associated with the formation or maintenance of an advice tie, which perhaps explains why the AIC and BIC values were higher in Model 4 than Model 3 (these variables do not help explain the dependent variable, and therefore create a less parsimonious model). However, Model 3 does not account for advice-seekers’ needs, which our theory suggests is an important determinant for advice-tie formation. Therefore, we chose to report on Model 4 in the main text because it includes all of our hypothesized variables.

Model 3 and Model 4 results (for both general and TF-CBT advice seeking networks) were similar with one exception. In the general advice-seeking network, sharing the same disciplinary field was not significant in Model 3. Yet, when the variables accounting for the advice-seeker’s needs were entered, being in the same disciplinary field was associated with general advice-seeking (b=0.329, SE=0.166).

Table 1. ERGM Model Building Results – Factors Associated with Formation and Maintenance of General Advice Ties

|  | Characteristic | Model 1 | | | Model 2 | | | Model 3 | | | Model 4 | | |
| --- | --- | --- | --- | --- | --- | --- | --- | --- | --- | --- | --- | --- | --- |
|  |  | est | | se | est | | se | est | | se | est | | se |
| edges (density) |  | -5.063 | * | (0.113) | -4.977 | * | (0.122) | -6.942 | * | (0.820) | -7.078 | * | (0.497) |
| General Ties Lag | Existing Relationships | 3.008 | * | (0.247) | 1.985 | * | (0.233) | 2.032 | * | (0.247) | 1.996 | * | (0.244) |
| Specific Ties Lag | Existing Relationships | 1.534 | * | (0.292) | 1.205 | * | (0.209) | 0.755 | * | (0.210) | 0.817 | * | (0.213) |
| mutual (reciprocity) | Existing Relationships | 2.460 | * | (0.383) | 1.655 | * | (0.300) | 1.851 | * | (0.312) | 2.083 | * | (0.353) |
| gwesp (transitivity) | Existing Relationships | 1.454 | * | (0.162) | 0.758 | * | (0.218) | 0.671 |  | (0.376) | 0.624 |  | (0.636) |
| gwesp.alpha | Existing Relationships | 0.829 | * | (0.108) | 1.497 | * | (0.115) | 1.643 | * | (0.161) | 2.164 | * | (0.149) |
| 3-cycles | Existing Relationships | -1.484 | * | (0.356) | -1.065 | * | (0.241) | -0.852 | * | (0.255) | -0.988 | * | (0.274) |
| Same Agency | Accessibility |  |  |  | 2.177 | * | (0.214) | 2.920 | * | (0.239) | 2.818 | * | (0.249) |
| Same Cohort | Accessibility |  |  |  | -0.342 | * | (0.166) | 0.199 |  | (0.175) | 0.265 |  | (0.194) |
| Same Field | Accessibility |  |  |  | -0.269 |  | (0.141) | 0.321 |  | (0.180) | 0.329 | * | (0.166) |
| Senior Lead Popularity | Expertise Quality (alter) |  |  |  |  |  |  | 0.074 |  | (0.289) | 0.321 |  | (0.301) |
| Supervisor Popularity | Expertise Quality (alter) |  |  |  |  |  |  | 0.887 | * | (0.225) | 0.822 | * | (0.226) |
| Expert Popularity | Expertise Quality (alter) |  |  |  |  |  |  | 4.088 | * | (0.291) | 4.185 | * | (0.305) |
| Training Popularity | Expertise Quality (alter) |  |  |  |  |  |  | -0.014 |  | (0.185) | 0.146 |  | (0.210) |
| Alter Higher Experience | Expertise Quality (alter) |  |  |  |  |  |  | 0.341 |  | (0.225) | 0.245 |  | (0.234) |
| Senior Lead Activity | Expertise Need (ego) |  |  |  |  |  |  |  |  |  | -0.381 |  | (0.306) |
| Supervisor Activity | Expertise Need (ego) |  |  |  |  |  |  |  |  |  | -0.072 |  | (0.233) |
| Training Activity | Expertise Need (ego) |  |  |  |  |  |  |  |  |  | -0.293 |  | (0.174) |
| Experience Activity | Expertise Need (ego) |  |  |  |  |  |  |  |  |  | 0.116 |  | (0.098) |
| AIC |  | -3157.9 |  |  | -3169 |  |  | -3438.4 |  |  | -3344 |  |  |
| BIC |  | -3103.6 |  |  | -3091.4 |  |  | -3306.5 |  |  | -3196 |  |  |
| *p<.05 |  |  |  |  |  |  |  |  |  |  |  |  |  |

Table 2. ERGM Model Building Results – Factors Associated with Formation and Maintenance of TF-CBT Specific Advice Ties

|  | Characteristic | Model 1 | | | Model 2 | | | Model 3 | | | Model 4 | | |
| --- | --- | --- | --- | --- | --- | --- | --- | --- | --- | --- | --- | --- | --- |
|  |  | est | | se | est | | se | est | | se | est | | se |
| edges (density) |  | -4.893 | * | (0.088) | -4.778 | * | (0.123) | -7.633 | * | (0.405) | -6.887 | * | (0.520) |
| Specific Ties Lag | Existing Relationships | 2.174 | * | (0.205) | 1.822 | * | (0.274) | 1.437 | * | (0.233) | 1.515 | * | (0.259) |
| General Ties Lag | Existing Relationships | 2.102 | * | (0.246) | 1.319 | * | (0.230) | 1.458 | * | (0.254) | 1.379 | * | (0.340) |
| mutual (reciprocity) | Existing Relationships | 1.485 | * | (0.409) | 0.788 |  | (0.410) | 1.166 | * | (0.392) | 1.213 | * | (0.420) |
| gwesp (transitivity) | Existing Relationships | 1.664 | * | (0.099) | 1.450 | * | (0.121) | 1.060 | * | (0.125) | 0.991 | * | (0.119) |
| gwesp.alpha | Existing Relationships | 0.613 | * | (0.078) | 0.470 | * | (0.102) | 0.908 | * | (0.104) | 0.847 | * | (0.115) |
| 3-cycles | Existing Relationships | -1.104 | * | (0.312) | -1.103 | * | (0.232) | -0.915 | * | (0.248) | -0.876 | * | (0.266) |
| Same Agency | Accessibility |  |  |  | 1.883 | * | (0.223) | 2.643 | * | (0.246) | 2.683 | * | (0.273) |
| Same Cohort | Accessibility |  |  |  | -0.355 | * | (0.165) | 0.202 |  | (0.196) | 0.256 |  | (0.215) |
| Same Field | Accessibility |  |  |  | -0.400 | * | (0.147) | 0.375 | * | (0.176) | 0.372 | * | (0.187) |
| Senior Leader Popularity | Expertise Quality (alter) |  |  |  |  |  |  | 0.074 |  | (0.296) | 0.187 |  | (0.370) |
| Supervisor Popularity | Expertise Quality (alter) |  |  |  |  |  |  | 1.047 | * | (0.206) | 1.092 | * | (0.223) |
| Expert Popularity | Expertise Quality (alter) |  |  |  |  |  |  | 4.236 | * | (0.327) | 4.242 | * | (0.305) |
| Training Popularity | Expertise Quality (alter) |  |  |  |  |  |  | 0.138 |  | (0.176) | 0.170 |  | (0.193) |
| Alter Higher Experience | Expertise Quality (alter) |  |  |  |  |  |  | 0.624 | * | (0.226) | 0.644 | * | (0.208) |
| Senior Leader Activity | Expertise Need (ego) |  |  |  |  |  |  |  |  |  | -0.288 |  | (0.306) |
| Supervisor Activity | Expertise Need (ego) |  |  |  |  |  |  |  |  |  | 0.075 |  | (0.226) |
| Training Activity | Expertise Need (ego) |  |  |  |  |  |  |  |  |  | -0.200 |  | (0.181) |
| Experience Activity | Expertise Need (ego) |  |  |  |  |  |  |  |  |  | 0.053 |  | (0.106) |
| AIC |  | -2910.4 |  |  | -2995.8 |  |  | -3335.9 |  |  | -3312.6 |  |  |
| BIC |  | -2856.1 |  |  | -2918.3 |  |  | -3204.0 |  |  | -3165.2 |  |  |

*p<.05
